# Psychological effects of water scarcity on community members: a case study of Lephalale municipality, Limpopo province, South Africa

**DOI:** 10.3389/fpsyg.2025.1537992

**Published:** 2025-02-04

**Authors:** Mmakwena Linda Seretlo-Rangata, Tholene Sodi, Saraswathie Govender

**Affiliations:** Department of Psychology, University of Limpopo, Polokwane, South Africa

**Keywords:** water, water scarcity, Lephalale municipality, water shortages, climate change, psychological effects

## Abstract

**Introduction:**

Water is essential for human survival and serves various purposes, including domestic, socioeconomic, and agricultural activities. However, water scarcity has emerged as a significant threat to this vital resource, posing a global challenge. While discussions surrounding the consequences of water scarcity typically emphasizes its effects on physical health and socioeconomic impacts, the mental and psychological effects on communities are often overlooked. Thus, the study aimed to explore the psychological effects of water scarcity on community members in Lephalale municipality, Limpopo province, South Africa.

**Methods:**

A qualitative research approach was used in the study. Participants were selected using purposive sampling and twenty participants who relied mainly on communal taps to access water were included (10 males and 10 females). Semi-structured in-depth interviews were used to collect data and interviews were audio recorded and later transcribed and translated by a language expert. Data were analyzed using content thematic analysis.

**Results:**

Three main themes emerged from the study results: (1) emotional distress; (2) interpersonal conflicts; (3) disruptions in the activities of daily living. Emotional distress included feelings of shame, embarrassment, anger, disappointment, hopelessness and helplessness. Additionally, interpersonal conflicts arose due to competition for water resources, and disruptions in daily activities were linked to water access challenges.

**Conclusion:**

The study findings demonstrated that water scarcity has a significant psychological impact on community members. The study concludes by recommending the integration of psychological principles and involvement of mental health care practitioners when developing water management programmes, strategies, interventions, and policy. The study can provide policy makers and mental health care practitioners valuable insights into the mental health challenges faced by communities affected by water scarcity. Moreover, these study findings can assist mental health care practitioners to tailor their interventions to address the specific needs of communities experiencing water scarcity, particularly community members presenting with mental health challenges associated with this issue.

## 1 Introduction

Water scarcity is one of the world's greatest challenges affecting the wellbeing of communities. Around 1.2 billion people worldwide reside in areas with shortage of water (Fan et al., 2014). Approximately 2.8 billion people worldwide lack access to clean drinking water (Hanjra and Qureshi, 2010). The scarcity of water is a concerning global risk, with the nature and the extent of the water crises varying from one country to another. Water scarcity involves two main phenomena: growing freshwater consumption and the depletion of available freshwater supplies, leading to water stress (Hedden and Cilliers, 2014; Schwerdtner Máñez et al., 2012). Water scarcity can occur when there is inadequate water to satisfy the demand. It can occur even regions that have abundant water resources where demand exceeds the supply capacity (Ibrahim and Mensah, 2017). In Africa, safe water resources are inaccessible to over 30% of the population as a result of the effects of global change on the availability of water (Varghese et al., 2013). There is a concern for future chronic and acute water scarcity in Africa which may be due to large population growth and climate change decrease in precipitation and runoff (McNally et al., 2019). There are already some subregions and countries in Africa that are experiencing growing water (Zhuwakinyu and Creamer Media, 2012). The major impacts of climate change in southern Africa are evident through water resources as witnessed in water insecurities. There is an anticipated decrease of about 20% in yearly precipitation by 2080 in the Southern African Development Community (SADC) which will lead to a reduction in water resources (Matchaya et al., 2019). Similar to most countries in the SADC, South Africa is facing a water crisis (Viljoen and Van der Walt, 2018) and was ranked as the 30th driest country globally with an average yearly rainfall of 450 mm which is below the world average of 860 mm (Hedden and Cilliers, 2014). The available water resources in South Africa are not equitably distributed and are sometimes used inefficiently. Thus, water is a precious resource (Zhuwakinyu and Creamer Media, 2012). Insufficient water infrastructure maintenance and investment, frequent prolonged droughts driven by climatic discrepancies, inequities in access to water and deteriorating water quality contribute to the water crisis in South Africa (Viljoen and Van der Walt, 2018; Kohler, 2016; Ziervogel et al., 2014). Failure to adequately invest in water services and to collect, treat and reuse water is exacerbating water shortages in many parts of the world (Zhuwakinyu and Creamer Media, 2012). Multiple cities across South Africa have reported experiencing significant water shortages (Muller, 2017; Pamla et al., 2021). Additionally, in Lephalale municipality, a generally water-scarce area, the villages (rural settlements) are the most acutely affected by the lack of water and a lot of the villagers are still compelled to fetch water from the taps outside their dwellings (Phadi and Pearson, 2018). Residents in many poorer communities experienced the burden of fetching and carrying water from outside their houses and were accustomed to living on < 50 L per person per day (Muller, 2018). Household surveys conducted in 2016 by Statistics South Africa (2017) suggested that not all members of rural communities had access to reliable and safe household water supplies. In some communities, despite the availability of water supply infrastructure, it is common for residents to experience extended periods without water flowing from the taps, particularly during hot weather seasons, sometimes lasting for months (Muller, 2018).

The crisis of water scarcity poses a significant threat on economic growth and on the wellbeing of everyone in South Africa (Viljoen and Van der Walt, 2018). Prior studies (Mushavi et al., 2020; Goldin, 2010; Khodarahimi et al., 2014a; Stevenson et al., 2016) found that community members experience increased bother and emotional distress due to altered water availability, water shortages and insecurity. Coêlho et al. (2004) reported that individuals in the areas with water shortages had significantly higher levels of emotional distress than individuals in the no-drought areas. There is a high prevalence of mental health problems, stress and worry are significantly higher in rural residents with water shortages. Rural citizens with drinking water shortages experience elevated levels of negative emotions than rural citizens who had no drinking water shortages (Khodarahimi et al., 2014b). Additionally, water scarcity and its related challenges are reportedly the key drivers of conflict situations amongst various communities (Gleick, 2014). In Sudan (Darfur), scarce water resources and a lack of access to water are one of the contributing factors of the ongoing conflicts (Smith, 2017). Furthermore, water scarcity interferes with other areas of people's lives. Opportunities for children to attend school are halted or interrupted, families are forced to migrate, increased time and effort is devoted toward getting water [UNICEF (United Nations Children's Fund), 2021]. It is evident from the literature above that water scarcity affects communities' overall wellbeing. Sadly, the typical focus of drinking water interventions is on promoting human health, reducing waterborne diseases and wellbeing while mental health in relation to water has received significantly less attention. The traditional water interventions are primarily designed to promote physical health and do not necessarily aim at reducing psychosocial distress (Stevenson et al., 2016; Thomas and Godfrey, 2018). Thus, a broad understanding of the psychosocial and mental distress related to water scarcity is necessary for developing comprehensive services that consider the mental health of water consumers (Toivettula et al., 2023). Furthermore, although prior studies have examined the various effects of water scarcity on communities, limited attention has been given to the impact of water scarcity on rural communities, particularly within the South African context. To address this gap, this study explored the psychological impacts of water scarcity on a rural community in Limpopo province, South Africa, offering a deeper understanding of the mental health challenges faced by the community as a result of water scarcity.

## 2 Material and methods

The current study employed a qualitative research approach. Qualitative research approaches allow the researcher to obtain an in-depth understanding of the thoughts and inner experiences of the participants through methods such as interviews (Corbin and Strauss, 2015; Creswell and Creswell, 2018). The current study sought to explore and gain in-depth understanding about the psychological effects of water scarcity on community members. Thus, a qualitative approach was deemed appropriate for the present study.

### 2.1 Study area

The study was conducted at Lephalale municipality, Limpopo province, South Africa in one of the rural tribal settlements, Ga-Seleka village. Lephalale municipality has encountered alarming incidences of water shortages. In certain cases, Lephalale municipality was required to deploy water tankers and install boreholes to ensure that every household had access to water within a 200 m radius of each dwelling. Nevertheless, despite these measures, significant challenges related to water shortages persist, exacerbated by the town's rapid growth and the proliferation of informal settlements in the surrounding areas (Lephalale Municipality, 2018).

### 2.2 Participants

Participants were selected using purposive sampling. In this study, purposive sampling was used to identify participants based on the researcher's judgment, selecting individuals with the most relevant knowledge about the phenomenon being studied (Creswell and Creswell, 2018; Babbie, 2014). A sample of 20 community members of Ga-Seleka village in Lephalale were selected. Age is a significant determinant of household water consumption behavior, with older individuals being more likely to prioritize the economical use of resources and practice water-efficient behaviors (Murwirapachena, 2021). Thus, the participants included 10 females and 10 males, aged 40–60 years. Additionally, all participants were community members who relied on communal taps as their primary source for water. According to the Lephalale Municipality (2014), one-third of households in this area lack access to water within their dwellings or yards, relying on community standpipes (Lephalale Municipality, 2014). Consequently, the participants were individuals who predominantly depended on communal taps to access water. The participants' demographic information is presented in Table 1.

**Table 1:** Participants' demographic information.

**Variable**	**N**	**%**
**Gender**	
Males	10	50%
Females	10	50%
**Age**	
40–50 years	08	48%
50–60 years	12	52%
**Water source**	
Communal taps	20	100%
Extra source of water	10	50

### 2.3 Data collection

Data were collected using semi-structured face-to-face interviews to elicit in-depth views and opinions from the participants (Creswell and Creswell, 2018). This method allowed for flexibility in probing participants' response, generating extensive and rich data from participants (Howitt, 2016). Data collection took place from October 2020 until April 2021. The interviews were scheduled in advanced and took place at the participants' preferred locations. All participants chose to have the interviews conducted in their homes and made prior arrangements to ensure limited interruptions. The interviews lasted from 25 to 40 min and covered key themes related to the psychological effects of water scarcity, interpersonal conflicts, and daily living disruptions. Data collection continued through an iterative and reflexive process continuing until the researchers determined that sufficient rich data had been gathered to address the research questions effectively (Braun and Clarke, 2020). After conducting 18 interviews, it became evident that no new information was emerging from the data. To confirm this, two additional interviews were carried out. In total, 20 interviews were conducted, with no follow-up interviews conducted with the participants. All interviews were audio-recorded with participants' consent and transcribed immediately using MS Word to enhance data accuracy. During the transcription process, codes were used to disguise participants' identifying information, ensuring protection of their privacy. Transcripts initially produced in Setswana and Sepedi were translated into English by a language expert to maintain the authenticity of the participants' expressions. The accuracy of translations was cross-checked to preserve cultural and contextual nuances.

### 2.4 Data analysis

Data were analyzed using the thematic content analysis following phases as recommended by Braun and Clarke (2006, 2014, 2020). The process of analysis was not linear; instead, it was conducted following a flexible iterative process, allowing for an in-depth exploration of patterns within the data. The initial step involved listening to the audiotapes and reading the transcripts multiple times to obtain a thorough insight and be familiar with the data. At this stage, the notes that were kept during data collection assisted in developing initial ideas about the data. Subsequently, the first author organized data through manual line-by-line coding, systematically making notes and highlighting texts to identify significant features and patterns within the data. Afterward, various initial codes were sorted and combined to develop emerging themes. Relevant extracts from the transcripts corresponding with the identified themes were then collated. Next, themes were reviewed and modified to ensure that a coherent story was developed from the data. Additionally, the first author maintained a track of codes and in collaboration with co-researchers, themes were reworded, restructured, some deleted or collapsed into other themes. Furthermore, individual themes were thoroughly analyzed, defined, and assigned clear and concise names along with brief descriptions to capture the story each theme conveys and its connection to the broader narrative of the research study. The final phase involved compiling a report that presented a concise and coherent narrative of the study. This included providing sufficient evidence for the identified themes through data extracts and contextualizing the findings within the context of existing literature. Throughout the process, the authors maintained a reflexive stance, acknowledging their influences of their perspectives on the analysis. Additionally, the authors prioritized regular team discussions to ensure a more nuanced and rigorous interpretation of the data.

### 2.5 Ethics

In accordance with the ethical research guidelines, ethical clearance was obtained from the University of Limpopo's Research Ethics Committee (TREC/151/2020:PG) prior to the commencement of the study. Permission to interview the community members was also granted by the local tribal authority, the Seleka Traditional Council. Additionally, the participants provided their written informed consent to participate in the study. Prior each interview, a brief overview of the study objectives and associated risks were explained to the participants (Khan, 2014; Neuman, 2011). To ensure confidentiality and privacy, codes were used to identify the participants instead of using their personal information or names (Fouka and Mantzorou, 2011).

## 3 Results

### 3.1 Emotional distress

The study findings indicated that water scarcity in the community cause substantial emotional distress amongst participants. In the current study, emotional distress is defined as a state of mental anguish triggered by a stressful event, encompassing a broad spectrum of emotions and psychological responses. The specific emotions and psychological reactions reported by the participants are elaborated in the following sub-themes:

#### 3.1.1 Stress, frustration, hopelessness, and helplessness

The study findings revealed that water shortages caused significant emotional distress, including stress, frustration, hopelessness and helplessness. Participants frequently expressed their struggles with the unreliability of water supply:

“*Sometimes we can go days, up to two to three days without access to water, imagine! Iyo!”* (P5).

This statement highlights the acute frustration stemming from prolonged interruptions in water availability. Consistently, the frustration was evident in the responses of other participants:

“*There is a major water shortage in our village. It is such a big problem. I wonder how are we supposed to live like this”* (P11).

The frustration regarding the ongoing water shortages in the community is illustrated by the participant's comment. Participants further expressed frustration over the financial burden resulting from water scarcity in the community. One participant stated:

“*In my house, we spent about R45 000 to drill boreholes and we didn't have a choice because there is no water in our area, Iyo! this is a lot of money”* (P14).

The statement above does not only underscore the frustrations experienced by community members, but it also highlights the limited options available to them, forcing them to drill boreholes due to water scarcity. These sentiments were echoed by other participants, who expressed feelings of hopelessness and helplessness over having no alternative but to invest in drilling boreholes:

“*On days when the government water is not available, we don't have a choice but to go to people who have boreholes and buy water from them. This requires us to have money to be able to get water, imagine that!”* (P20).

This assertion further highlights the frustration associated with the financial burden of securing water. This viewpoint was echoed by another participant:

“*Water is so scarce in our community. It is very costly to drill a borehole but what choice do people have?”* (P10).

This statement reflects both frustration with the ongoing scarcity and a sense of helplessness about the lack of solutions to address the issue. These emotions were consistent across participants, many of whom described feeling overwhelmed by the persistent challenge of securing water for daily needs. For some, this stress translated into feelings of despair about their living conditions and the uncertainty of improvement. While stress and frustration were immediate responses to water shortages, participants also reported long-term emotional tolls, including feelings of hopelessness about their future in the village. These findings suggest that the psychological impact of water scarcity extends beyond short-term distress, influencing participants' overall mental health and wellbeing.

#### 3.1.2 Shame and embarrassment

The study findings showed that participants felt ashamed and embarrassed by the impact of water shortages on their lives:

“*We can go about two weeks without doing the washing because water is very scarce. Imagine, not washing your clothes, how embarrassing is that”* (P10).

The participants experienced embarrassment for going days without attending to basic needs, such as doing laundry, due to water shortages. Another participant added that:

“*Sometimes the municipality brings us water tanks but ques are very long and you wait for long periods just to get water. It's a shame on us, we are struggling”* (P 13).

This statement illustrates that waiting in long queues to get water causes feelings of shame among community members. Overall, the study findings suggest that shame and embarrassment experienced by the community stem not only from the negative impact of water scarcity on their personal hygiene but also from the extended time spent sourcing water.

#### 3.1.3 Disappointment and anger

The study findings revealed a sense of disappointment resulting from unmet expectations and unfulfilled promises made by authority figures regarding water provision in the community. Participants expressed their despondency over the ongoing struggle to access water:

“*This issue of water shortages has been reported multiple times to the municipality and they promised to help. It's disappointing because we still struggle”* (P13).

This statement illustrates the participants' disappointment with the municipality's lack of response, despite repeatedly reporting water scarcity issues in the community. Moreover, similar sentiments of disappointment were echoed by several other participants. One participant stated that:

“*We struggle a lot with water and we travel long distances to reach taps that have water. This is so disappointing that even now, our people struggle to get access to water”* (P7).

The disappointment felt by the participants is further exacerbated by walking long distances to access water, even in the modern era. Another participant expressed that:

“*The population keeps growing but water infrastructure remains the same size which can't accommodate the whole village* (P11).

This participant underscores a critical concern regarding the imbalance between the community's ongoing growth and the stagnant state of the water infrastructure, which is insufficient to support the expanding population. Additionally, one participant expressed anger about the inequitable distribution of water in the community:

“*Only the old villages were supplied with water pipes and now this creates a problem for the new villages. It makes me angry because it's unfair for other people to have access to water while others don't”* (P7).

The study findings indicate that community members are disappointed with local authorities over various factors contributing to water scarcity, such as the outdated and poorly maintained water infrastructure that is unable to meet the growing community's current water demands. Additionally, the community's feelings of anger were related the unfair and unequal distribution of the already scarce water resources.

### 3.2 Interpersonal conflicts

The study findings indicated that water shortages in the community contributed to interpersonal conflicts both amongst community members and with neighboring villagers. In the context of this study, interpersonal conflicts refer to disagreements or disputes between individuals, primarily arising from competition over scarce resources, such as water.

#### 3.2.1 Intragroup conflicts

The findings of the study revealed that water shortages contribute to interpersonal conflicts among members of the same village. Participants' responses highlighted that; relationships are strained as individuals compete for the scarce water resources:

“*Sometimes government supply water by bringing water tanks. Sometimes people fight for this water”* (P5).

The participants often engage in conflicts over the limited water resources provided by the government in response to water scarcity. The comment above reveals that when the government supplies water tanks to the community, disputes frequently arise as individuals compete to secure access to the water. Additionally, some participants expressed feelings of frustration and dissatisfaction, attributing their inability to access water to issues such as theft and the misuse of communal water infrastructure, including taps:

“*… we don't get access to water fairly and people often think selfishly by stealing those communal taps. Tell me why would someone steal a tap that helps the whole community”* (P16).

It appears that there is a growing irritation and frustration directed toward individuals who prioritize their own needs over those of the community by stealing communal water infrastructure, such as taps. Another participant expressed similar sentiments regarding community members who consistently prioritize the own needs when it comes to water access:

“*We don't share water equally. Some people use pressure pumps on communal taps. The water runs out before others can get access to it, because of these pressure pumps. This is really irritating!”* (P15).

This assertion indicates frustration and aggravation toward community members who use pressure pumps, further limiting equitable access to water. Regrettably, the aggravation expressed in the sentiments above could lead to conflicts and potentially damaging the quality of relationships amongst community members, as well as create deeper social divisions. The study findings show that conflicts among community members arise from disputes over the equal sharing of water. For some participants, these conflicts stemmed from fighting for the water provided by the government in response to the community's water scarcity. For others, the conflicts were fueled by frustrations over community members prioritizing their own needs over the collective good when it came to accessing water. The findings highlight the growing tensions and divisions within the community, exacerbating existing issues of water scarcity and inequality.

#### 3.2.2 Intergroup conflicts

The study findings also revealed that interpersonal conflicts emerged among neighboring villages. The intergroup conflicts suggest that disputes over access to water extended beyond individual households in the community to include inter-village tensions. Participants expressed dissatisfaction regarding the access to water from their communal taps by individuals from neighboring villages:

“*There are people from neighboring villages also struggling with water who come get water from water taps on the streets when we have water. We can't share with them as we are also struggling with water shortages. It might sound bad, but actually, we can't share with them”* (P9).

This statement highlights the participants' dissatisfaction with neighboring villagers accessing water from their communal taps. Consistent with this assertion, another participant added that:

“*People from the new village walk down toward our side of the village to get water from our communal taps that have water. This is not fair at all”* (P18).

These assertions emphasize the frustration community members experience regarding other individuals from other villages accessing water from their communal taps. This frustration could potentially escalate into conflicts between the community members and individuals from neighboring villages, as competition for limited water resources intensifies. The study findings demonstrate that water scarcity not only poses a challenge to water availability but also strains social dynamics, fostering disputes and tensions over the equitable allocation and access to water.

### 3.3 Disruption of daily activities of living

The study findings indicated that water shortages within the community disrupted the participants' daily activities of living (DALs). In this context, the daily activities of living refer to routine tasks that individuals perform throughout the day including work, social interactions, self-care, and household maintenance.

#### 3.3.1 Effects of personal hygiene

The study findings indicate that water shortages impact overall hygiene practices, including bathing and personal grooming. One participant explained that:

“*Our hygiene is affected. Currently we are getting a bit of rain, we are busy doing their laundry, even old clothes from December and blankets from last year's winter. We are only washing them now”* (P13).

This participant raises a concern regarding the impacts of water scarcity on the personal hygiene of community members, as they often go for long periods without being able to do their laundry, which compromises their hygiene. This was corroborated by another participant, who stated that:

“*Our hygiene is also compromised because we can't clean and wash our clothes”* (P16).

This assertion further shows water scarcity affects the community members ability to maintain good personal hygiene. The study findings reveal that, at times, community members rely on rainwater to carry out essential hygienic activities, such as doing laundry. This highlights the severity of water shortages in the community and its significant impact on the overall hygiene of community members, making it difficult for them to maintain basic cleanliness.

#### 3.3.2 Effects on other daily activities (social, occupational)

Based on the findings of the study, the daily routines, schedules, and chores of community members were significantly disrupted by recurring water shortages. One participant indicated that:

“*Imagine all the time we are spending trying to get water, we could be doing something productive with that time”* (P13).

This participant complained that people in the community are spending prolonged periods trying to get water, which interferes with their ability to engage in other activities. Another participant further remarked that:

“*Imagine, pushing the wheelbarrow for so long and spending so much time just to get water”* (P18).

From the assertions above, it is apparent that spending prolonged periods fetching water and traveling long distances to collect water from other areas of the village caused significant disruptions to the participants' daily routines and schedules. This not only consumed valuable time but also affected their ability to engage in other important activities.

## 4 Discussion

### 4.1 Emotional distress

The study results revealed that participants were stressed, frustrated and felt hopeless about water scarcity in the community. Furthermore, unmet expectations and unfulfilled promises by local authority figures appear to exacerbate these feelings experienced by participants. Similarly, Khodarahimi et al. (2014b) found that the prevalence of mental health problems, stress and worry is significantly higher in rural residents with water shortages. Prolonged drought is a serious stressor for rural communities and may lead to emotional distress, worry and increased irritability (Sartore et al., 2007). Consistently, Khodarahimi et al. (2014a) added that emotional reactions to water shortages in rural regions include anguish, pessimism, mental distress, anger and irritability, guilt, and being overwhelmed by negative emotions. Furthermore, participants expressed frustration regarding the financial effects of water scarcity. The financial effects expressed by participants are mostly related to the costs of drilling boreholes and buying water from people who have boreholes. Consistent with the study findings, Thomas and Godfrey (2018) found that emotional distress regarding the water-related issues was linked to the cost of getting water. This is because many homes in Southern Africa still do not have piped-in water, so household members have to get water from standpipes, buy it from vendors or incur the costs of drilling boreholes. Thus, a greater proportion of household income may need to be spent on water delivered from private sources, such as tankers, to supplement the lack of water locally (Intergovernmental Panel on Climate Change, 2001; Majuru, 2015; Ziervogel, 2018).

The findings of the study further showed that participants experienced feelings of shame associated with traveling long distances and queueing for longer periods to access water. The feelings of embarrassment appeared to also be related to the effects of water scarcity on the participants' daily activities such as grooming and bathing. These supported the findings by Bulled (2017) who found that people felt distressed and embarrassed about their water shortage situations and regarding wasting time or money to acquire water. Hove et al. (2019) further found that water shortages were a significant source of personal unhappiness, stress and embarrassment for community members who had to continually collect water. Consistently, Tshabatau (2020) found that women in Kweneng district (Botswana) have experienced feelings of embarrassment due to altered bathing routines and skipping showers as a result of water shortages. Bulled (2017) further found that people felt distressed, shame and embarrassment about their water shortage situations and concerning money or wasting time to acquire water.

Feelings of anger about the unfair distribution of available water amongst the community were also apparent. The community members' inability to receive water due to unmaintained, old water infrastructure as well as theft of water infrastructure appeared to worsen the feelings of anger and disappointment. In line with the study findings, Pamla et al. (2021) reported that people attributed water scarcity in Makhada (South Africa) to the municipality's failure, to invest in the upkeep and extension of current water infrastructure in the face of expanding population and water demand. Furthermore, it appeared that the lack of proper maintenance of water systems and infrastructure by authorities in most places altered water availability in ways that increased bother and distress (Khatri and Vairavamoorthy, 2007; Mushavi et al., 2020; Pearson et al., 2015; Pamla et al., 2021). Consistently, Taing et al. (2019) further reported that the residents of Cape Town lashed out in anger at the City for focusing on implementing water-use behavior interventions instead effectively planning for the severe drought from 2015 to 2018. The study results further indicated that feelings of disappointment are also associated with the illegal connection of water infrastructures such as private pipes and the theft and damage of water taps and caps on communal taps by other community members. Similarly, Peal et al. (2014) expressed a concern that the acts of vandalism and theft of valuable water supply tools such as metal pipes and fittings lead to increased operation and maintenance costs (that is, repair or replacement of vandalized pipes) and reduced access to a suitable quantity and quality of water. Unauthorized water connections cause a lot of water loss and affect the provision of water to households that are connected formally (Theodory and Ndunguru, 2013).

### 4.2 Interpersonal conflicts

It emerged from the participants' narratives that interpersonal conflicts arise amongst community members from the negotiations regarding sharing the available water. In agreement with the findings of the study, Hove et al. (2019) found that there was an increase in conflicts and tension amongst households over mobile water tankers and who gets water first. Mushavi et al. (2020) further indicated that excess water demand at public water sources frequently caused verbal and physical disputes and conflicts among people in the queue. Moreover, Pamla et al. (2021) reported that the disproportionate impact of water scarcity on vulnerable groups can be a source of conflicts amongst communities. More previous literature report that water scarcity contributes to conflicts amongst communities (Bulled, 2017; Gleick, 2014; Smith, 2017).

The study findings indicated that conflicts also arise between community members and neighboring villagers who occasionally access water from their communal taps. Similarly, Mukuhlani and Nyamupingidza (2014) found that in Bulawayo (Zimbabwe) water restrictions and shortages led to conflicts as residents from Nketa 9 flocked to Nketa 7 with containers to secure water. Additionally, Pearson et al. (2015) noted that water scarcity and insecurities may precipitate interpersonal conflicts over water, between users at the local and regional levels within countries. Furthermore, United Nations Water (2007) echoed these findings by reporting that water shortages may worsen the conflict in existing water-stressed areas among local communities competing locally for access to natural springs and rivers, as well as lead to conflicts on a larger international transboundary scale between countries sharing a very limited and essential resource.

### 4.3 Disruption of daily activities of living

The study results showed that water scarcity affects the participants' activities of daily living. It was apparent from the participants' narratives that their daily activities of living such as bathing, cleaning, washing, grooming and social activities are affected by water shortages. Similar to study results, Tshabatau (2020) found that bathing and showering routines for women in Kweneng district were altered as a result of water shortages in the area. Moreover, Mushavi et al. (2020) reported that water insecurity made it difficult for some women to maintain good hygiene, which potentially jeopardized their standing with others in the community.

The findings of the study further showed that water shortages disrupt people's schedules and other activities (e.g., social and economic). Fetching water from far water resources disrupted community members' daily activities, took over people's daily schedules and disorganized their chores (Mushavi et al., 2020; Hove et al., 2019; UNICEF (United Nations Children's Fund), 2021). Likewise, Ziervogel (2018) pointed out that the time people spent fetching water could be spent on things like going to school or engaging in economic activities. Wutich (2006) also found that people's sociability decreases as water scarcity worsens during the dry season. Water shortages cause a major lifestyle disruption particularly in low-income households as reservoirs in low-income areas doesn't have capacity to supply water for the growing demand due to increasing population (Pamla et al., 2021; UNICEF (United Nations Children's Fund), 2021).

## 5 Limitations of the study

The current study has several limitations, one of which is the inclusion of only older participants. While there was a clear rationale for focusing on this demographic, this limitation suggests that the findings may not fully represent the broader population of individuals from various age groups who depend on communal taps in Lephalale, thereby reducing the generalizability of the results. Furthermore, the study did not account for demographic factors such as participants' educational and socio-economic status, as well as gender, during the analysis and interpretation of the findings.

## 6 Conclusion

The study aimed to exploring the psychological effects of scarcity on community members in Lephalale Municipality, South Africa. Like many countries worldwide, South Africa faces a significant challenge regarding water scarcity. Discussions surrounding water shortages often focus primarily on their impact on physical health, with less attention given to their psychological effects on communities. Through a comprehensive thematic content analysis, the study revealed that residents of Lephalale experienced various psychological difficulties as a result of water scarcity. The findings highlighted that these individuals faced emotional distress related to the scarcity of water. Moreover, the study indicated that water shortages led to interpersonal conflicts both within the community and with individuals from neighboring villages. Additionally, the study found that water scarcity disrupted the daily activities and routines of community members. The emphasizes the importance of integrating psychological insights and involving mental health care practitioners in the development of water management programmes, strategies, and interventions.

## Data Availability

The original contributions presented in the study are included in the article/supplementary material, further inquiries can be directed to the corresponding author.
